# Editorial: Extracellular vesicles: emerging roles in the aged and neurodegenerative brain

**DOI:** 10.3389/fncel.2024.1522499

**Published:** 2024-12-09

**Authors:** Foteini Vasilopoulou, Jennifer Pocock, Gal Bitan, Dirk M. Hermann

**Affiliations:** ^1^Department of Neuroinflammation, UCL Queen Square Institute of Neurology, University College London, London, United Kingdom; ^2^Department of Neurology, David Geffen School of Medicine, Brain Research Institute, and Molecular Biology Institute, University of California, Los Angeles, Los Angeles, CA, United States; ^3^Department of Neurology, University Hospital Essen, University of Duisburg-Essen, Essen, Germany

**Keywords:** extracellular vesicles, central nervous system, peripheral nervous system, neurodegeneration, neuroinflammation, neuroprotection, biomarkers

Over the past few decades, central nervous system (CNS) and peripheral nervous system (PNS) secreted nanosized extracellular vesicles (EVs) have emerged as promising biological platforms for advancing our understanding of intercellular communication, molecular transfer, and mechanisms underlying neurodegenerative and other CNS or PNS diseases (Mallach et al., 2021a,b; Li et al., 2022; Hermann et al., 2024). EVs are released by virtually all cells in the CNS and PNS and carry an heterogeneous cargo of proteins, DNAs, RNAs, metabolites, and lipids, that varies reflecting the state and origin of their parent cells. Once released into the extracellular space, EVs modulate cellular responses in the recipient cells to promote protective effects yet sometimes, inadvertently propagate toxic signals in a context-dependent manner. Importantly, their ability to deliver functional cargo, cross biological barriers, and reflect the state of their parent cells makes them promising candidates for therapeutics, biomarkers, and drug delivery systems (Bravo-Miana et al., 2024; Hermann et al., 2024). In this expanding field, researchers are increasing efforts to address scientific gaps related to the mechanisms regulating cargo loading, secretion and uptake, as well as the intra- and intercellular pathways triggered by EVs in disease, with the aim of enhancing their potential for clinical application. This Research Topic features two original studies and two reviews that provide new insights into the mechanisms by which EVs contribute to nervous system disease progression, opening avenues for therapeutic strategies and diagnostic advancements in neurological disorders.

The diseased CNS is often characterized by the presence of neuroinflammation, a process orchestrated by glial cells, including astrocytes and microglia, that relies on dynamic glia-glia or glia-neuron crosstalk. Such communication among cells occurs either through direct cell-cell contact or via cellular secretome products (Bernaus et al., 2020; Matejuk and Ransohoff, 2020; Middeldorp et al., 2023). Increasing evidence indicates that glia-released EVs can transmit inflammatory signals among CNS cells either fuelling the vicious cycle of inflammation (Gupta and Pulliam, 2014) or promoting inflammatory resolution (Markoutsa et al., 2022) through molecular mechanisms that remain an active area of research. Memo et al. explored the role of a subclass of CNS-released EVs, identified as small EVs (sEVs), in regulating CNS local tissue reactivity to inflammatory stimulus and inflammation spreading, with implications for neurodegenerative diseases, or any disease characterized by neuroinflammation. Using spinal cord organotypic cultures to mimic CNS resting and lipopolysaccharide (LPS) induced reactive states the authors exposed naïve (so far healthy) slices to sEVs released by resting or reactive spinal resident cells. They show that reactive sEVs induce the secretion of proinflammatory cytokines and chemokines, alter microglia morphology and trigger aberrant astrocyte calcium dynamics in naïve slices. Importantly, their results led to the proposal that the recruitment of naïve astrocytes by inflammatory sEVs is associated with increased astrocytic hemichannel permeability. Further supporting this, they observe that by specifically blocking connexin43 hemichannels expressed in sEVs, the inflammatory response in naïve slices is prevented; however, the underlying mechanisms need to be elucidated. These findings not only build upon the foundation laid by previous studies on EVs' critical role in propagating neuroinflammation, but also introduce a specific interaction between EV hemichannels and astrocyte membrane that has the potential to shift the understanding of the molecular mechanisms whereby sEVs mediate their effects to recipient cells.

As mentioned earlier, a key aspect in EV biology and potential for clinical application as biomarkers of CNS pathologies is their ability to mirror the state and content of the parental cells. Therefore, to fully unlock their potential, it is essential to use well-characterized and reliable cellular models that closely recapitulate human complexity. In this regard, modeling human CNS diseases in cell culture remains a challenging yet active area of research. Progress in this field should be regularly considered by researchers in the EV field to ensure that preclinical findings are translatable to clinical practice. White et al. address concerns arising from the prolonged use of serum when using cultured human astrocyte, and the reliability of the resulting astrocyte-derived EVs as per their cargo and state. The authors compare human astrocytic cultures in serum-containing and serum-free conditions and identify differences in morphology, astrocytic marker expression, proinflammatory cytokine release, transcriptome, and proteome of the cells, suggesting differences in the content and the reactive phenotype of the secreted EVs. According to this study, serum-free astrocytes are more akin to *in vivo* human resting astrocytes whereas upon chronic or acute exposure to serum, cultured astrocytes adopt a phenotype closely resembling human reactive astrocytes. Not surprisingly, corresponding proteomic changes are also identified in the EVs released by serum-containing (reactive) or serum-free (resting) cultured astrocytes. Particularly, EVs secreted by serum-cultured (reactive) astrocytes are smaller in size, can reactivate serum-free (resting) astrocytes, and their proteome is enriched in complement and coagulation cascade-related proteins that may propagate a neurotoxic effect to recipient cells. In contrast, the EV proteome from serum-free, resting astrocytes is enriched in proteins involved in synapse-related and homeostatic pathways suggesting a protective or supportive effect upon their uptake by neuronal or non-neuronal cells in the CNS. Collectively, the results of the study provide insights into astrocytic EV contents and signaling in the nervous system with implications for their use as biomarkers of CNS pathologies. Additionally, this study emphasizes the importance of carefully selecting cell-culture models for studying EV functions.

Beyond their detrimental role in driving inflammation and neurotoxicity in CNS diseases, CNS-derived EVs also serve as messengers of beneficial signals by delivering bioactive molecules to recipient cells (Mallach et al., 2021b). Exploiting their ability to cross the blood-brain-barrier (BBB) while safeguarding their contents has led to promising EV-based therapeutics that offer a novel strategy for treating neurological disorders. In the mini review by Chen et al., the authors take a focused approach to the therapeutic potential of exosome (an EV subclass) -based treatments for traumatic brain injury (TBI) and stroke with a special focus on recent advancements in engineered exosomes with an amplified therapeutic efficacy. The authors first highlight the critical role of exosomes in the protective paracrine effects delivered by cell-based therapies against TBI or stroke describing their properties and functions in the injured brain. Importantly, they provide a detailed examination of current findings showing neurorestorative and neuroprotective effects delivered by mesenchymal stromal cell (MSC)-secreted, neural stem cell-secreted or microglia-secreted exosomes in preclinical models of TBI and stroke, suggesting a potential neurorestorative therapy based on exosomal treatments. Moreover, the authors point out evidence showing that protective effects observed by exosomes in TBI and stroke experimental models are attributed to specific microRNAs (miRNAs), present in the exosomal cargo. Based on this evidence, they introduce the reader to the concept of engineered exosomes enriched with specific protective miRNAs. The authors postulate that modulating miRNA-related cargo in engineered exosomes may enhance treatment efficiency and amplify their therapeutic effects.

While much attention has been devoted to EVs secreted by brain and spinal cord cells and their role in complex CNS diseases, EVs released by PNS cells also have gained significant interest attributed to the regenerative properties of these EVs (De Jong et al., 2014; Hercher et al., 2022). The comprehensive review by Izhiman and Esfandiari focuses on peripheral nerve regeneration (PNR) mechanisms and the emerging role of EVs released in the PNS. The authors offer a detailed overview of the three-stage cascade of events following peripheral injury and the cell types involved in these processes, including Wallerian degeneration and myelin clearance mediated primarily by macrophages and Schwann cells (SCs), axonal outgrowth and regeneration facilitated by fibroblasts and SCs, and nerve remyelination supported by SCs. The authors highlight a crucial role of EVs, and specifically the subclass of sEVs, in facilitating cellular communication within the regenerative cascade. Particularly, the authors provide a detailed account of evidence demonstrating that sEVs released by macrophages, SCs, or fibroblasts mediate macrophage-nerve, SC-nerve, or SC-fibroblast communication, which in turn influences myelin clearance, axonal elongation, or myelin regeneration. The authors conclude that the impact of sEVs on these processes is attributed to distinct proteins and miRNAs associated with each cellular phenotype providing a detailed summary of distinct miRNAs identified as regulating these biological processes. Lastly, the authors delve into the latest findings concerning the therapeutic utilization of electrical stimulation and mechanotherapy, including ultrasound stimulation and extracorporeal shock wave by promoting regeneration within the PNS, along with the exploration of the associated signaling pathways that mediate the regenerative effects, which are shown to be intimately linked to increased secretion and uptake of sEVs. However, the exact mechanisms of release, uptake or cargo transportation of sEVs in PNR needs further exploration and future work could unravel the potential of sEVs as tools for studying intercellular communication during regeneration and EV-based regenerative treatments.

## Take home messages of this Research Topic and future directions

There are four main take home messages of this Research Topic, which have implications for future studies: (i) There is a need for continuous in-depth studies on EV-mediated mechanisms in CNS diseases which could not only provide valuable insights into disease pathways but may also uncover novel therapeutic targets. Importantly, studying single EVs extracted from biological fluids or tissues can reveal how EVs from specific cell types influence the progression of CNS diseases and identify EV signatures for diagnostic tools or targeted therapies. (ii) Special attention must be given to the appropriate selection of experimental models when studying EV biology and function, and to the methodology used for EV isolation, purification, and characterization to overcome challenges that limit the clinical translation of preclinical findings. (iii) Future research should address the mechanisms of EV transcytosis across the BBB, how EVs influence BBB function and properties and how these processes are affected in CNS diseases, which are often characterized by BBB damage. (iv) Studies on the role of EVs in neurodegenerative diseases should extend beyond the nervous system, as EVs may serve as key mediators of nervous system communication with peripheral systems.
